# Giorgio Maggioni master of pediatrics and the SIP’s group of History of Pediatrics

**DOI:** 10.1186/1824-7288-40-S1-A30

**Published:** 2014-08-11

**Authors:** Maria G Gregorio, Francesco S Biagiarelli, Luigi Cataldi

**Affiliations:** 1 UOCC ASL 8, Cagliari,09100, Italy; 2 La Sapienza University, Rome, 00100, Italy; 3 Dept. of Mother and Child, Division of Paediatrics, Catholic University of the Sacred Heart, Rome,00168, Italy

## 

**Figure 1:**
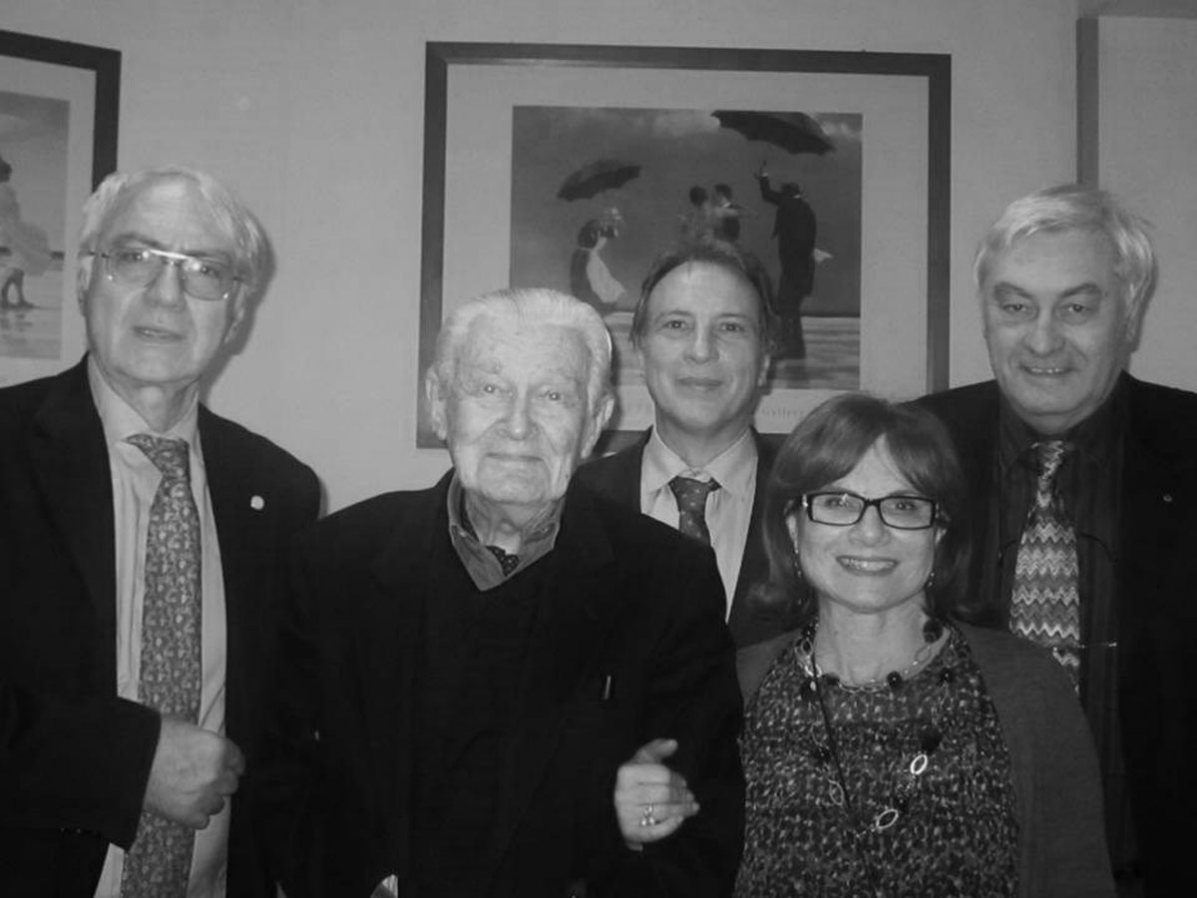
Group of History of Pediatrics, from left to right, Cataldi; Maggioni; Paladini; Gregorio; Fasani (26/2/2011).

It is limited for us, the old group of History of Pediatrics (Figure 1) (founded in 1998 in Turin thanks to Carlo Montinaro’s tenacity), remember the birth, death, prizes and scientific publications of prof. Giorgio Maggioni.

Is not a coincidence that the poster we presented at SIP’s congress in Palermo, has a title and different subtitles. This is because on the one hand we like to remember the prof. Maggioni’s figure, recently deceased (1/5/2014), and there you will find a short curriculum, and on the other hand we want to highlight the different sides of his personality.

Although it is difficult summarize in 400 words the Giorgio Maggioni’s scientific and cultural heritage, authors refer particularly to the Master’s commitment since 60th SIP’s congress in Turin (1998) in supporting our “newborn” group of History of Pediatrics.

Giorgio Maggioni had never been in the executive committee board, but he was the soul, the historical memory(1), the Honorary President, the Example.

"We must remind... people exists only in the memory", he said. And consequently, the interest, attachment for books so much to imagine the Paradise, as a big library with Mozart’s music in the background, books that kept him company and that he preferred on the television. We will always remember his intimate and tender side, in the role of father, example of intellectual honesty, curiosity for knowledge, but also liberal in letting choose the right way to each son, according to personal interests, or loving grandpa, that accepting grandchildren on his knees, invited them not to waste time, and to protect neurons from the decay.

And still “What you know no one can ever take away, remember that!”; “Knowledge is power” he added. He was a very tenderhearted person, always ready to hear you and give good advices, he never judged you. As Francesco recalls, his grandson pediatrician: the day of my graduation, he, seated and serious…but happy said: “I could not miss this historic event!”

He said: ”I wish you to be loved by your sons as i feel loved by mine”. In his memory, we want underline his clarity, childlike curiosity like he saw the world, medicine and history.

At last he always said ”I hope to see you soon”.

“Goodbye Master"

